# Possible Role of IRS-4 in the Origin of Multifocal Hepatocellular Carcinoma

**DOI:** 10.3390/cancers13112560

**Published:** 2021-05-23

**Authors:** Luis G. Guijarro, Patricia Sanmartin-Salinas, Eva Pérez-Cuevas, M. Val Toledo-Lobo, Jorge Monserrat, Sofía Zoullas, Miguel A. Sáez, Miguel A. Álvarez-Mon, Julia Bujan, Fernando Noguerales-Fraguas, Eduardo Arilla-Ferreiro, Melchor Álvarez-Mon, Miguel A. Ortega

**Affiliations:** 1 Unit of Biochemistry and Molecular Biology (CIBEREHD), Department of System Biology, University of Alcalá, 28801 Alcala de Henares, Spain; patricia.sanmartins@uah.es (P.S.-S.); e.p.cuevas@csic.es (E.P.-C.); eduardo.arilla@uah.es (E.A.-F.); 2 Ramón y Cajal Institute of Sanitary Research (IRYCIS), 28034 Madrid, Spain; jorge.monserrat@uah.es (J.M.); miguelangel.alvarezm@edu.uah.es (M.A.Á.-M.); mjulia.bujan@uah.es (J.B.); melchor.alvarezdemon@uah.es (M.Á.-M.); miguelangel.ortega@edu.uah.es (M.A.O.); 3 Unit of Cell Biology, Department of Biomedicine and Biotechnology, University of Alcala, 28871 Alcala de Henares, Spain; mval.toledo@uah.es; 4 Department of Medicine and Medical Specialities, Faculty of Medicine and Health Sciences, University of Alcalá, 28801 Alcala de Henares, Spain; sofiazoullas@alumni.harvard.edu (S.Z.); msaega1@oc.mde.es (M.A.S.); 5 Pathological Anatomy Service, Central University Hospital of Defence-UAH Madrid, 28801 Alcala de Henares, Spain; 6 University Center for the Defense of Madrid (CUD-ACD), 28047 Madrid, Spain; 7 Department of Surgery, Medical and Social Sciences, Faculty of Medicine and Health Sciences, University of Alcalá, 28801 Alcala de Henares, Spain; fernando.noguerales@uah.es; 8 Department of General Surgery, Principe de Asturias Hospital, 28871 Alcala de Henares, Spain; 9 Immune System Diseases-Rheumatology, Oncology Service an Internal Medicine, University Hospital Príncipe de Asturias, (CIBEREHD), 28806 Alcala de Henares, Spain; 10 Cancer Registry and Pathology Department, Hospital Universitario Principe de Asturias, 28806 Alcala de Henares, Spain

**Keywords:** nuclear IRS-4, PI3K, hepatocellular carcinoma, β-catenin, cyclin D, integrin α2/β1 and FAK

## Abstract

**Simple Summary:**

Hepatocellular carcinoma (HCC) is a potentially deadly liver cancer with a high prevalence worldwide. Despite the very efforts placed on this cancer, most cases are associated with poor prognosis and the understanding of the molecular mechanisms implicated in the development of HCC are arising as a potential therapeutic approach of this cancer. In this sense, we aimed to evaluate the established role of insulin receptor substrate 4 (IRS-4) in the tumorigenesis and progression of HCC. Thus, we leaded a histopathological study of this component, along with additional cancer biomarkers such as PCNA, Ki67, and pH3. In addition, in vitro models of different cell lines were used to describe the effects of IRS-4 overexpression/silencing. Finally, immunoblot analysis and transfection experiments were also conducted. Our research demonstrates that IRS-4 is involved in multiple tumoral effects such as proliferation, cell migration, and cell-collagen adhesion as well as the appearance of multifocal HCC.

**Abstract:**

New evidence suggests that insulin receptor substrate 4 (IRS-4) may play an important role in the promotion of tumoral growth. In this investigation, we have evaluated the role of IRS-4 in a pilot study performed on patients with liver cancer. We used immunohistochemistry to examine IRS-4 expression in biopsies of tumoral tissue from a cohort of 31 patient suffering of hepatocellular carcinoma (HCC). We simultaneously analyzed the expression of the cancer biomarkers PCNA, Ki-67, and pH3 in the same tissue samples. The in vitro analysis was conducted by studying the behavior of HepG2 cells following IRS-4 overexpression/silencing. IRS-4 was expressed mainly in the nuclei of tumoral cells from HCC patients. In contrast, in healthy cells involved in portal triads, canaliculi, and parenchymal tissue, IRS-4 was observed in the cytosol and the membrane. Nuclear IRS-4 in the tumoral region was found in 69.9 ± 3.2%, whereas in the surrounding healthy hepatocytes, nuclear IRS-4 was rarely observed. The percentage of tumoral cells that exhibited nuclear PCNA and Ki-67 were 52.1 ± 7%, 6.1 ± 1.1% and 1.3 ± 0.2%, respectively. Furthermore, we observed a significant positive linear correlation between nuclear IRS-4 and PCNA (r = 0.989; *p* < 0.001). However, when we correlated the nuclear expression of IRS-4 and Ki-67, we observed a significant positive curvilinear correlation (r = 0.758; *p* < 0.010). This allowed us to define two populations, (IRS-4 + Ki-67 ≤ 69%) and (IRS-4 + Ki-67 > 70%). The population with lower levels of IRS-4 and Ki-67 had a higher risk of suffering from multifocal liver cancer (OR = 16.66; CI = 1.68–164.8 (95%); *p* < 0.05). Immunoblot analyses showed that IRS-4 in normal human liver biopsies was lower than in HepG2, Huh7, and Chang cells. Treatment of HepG2 with IGF-1 and EGF induced IRS-4 translocation to the nucleus. Regulation of IRS-4 levels via HepG2 transfection experiments revealed the protein’s role in proliferation, cell migration, and cell-*collagen adhesion*. Nuclear IRS-4 is increased in the tumoral region of HCC. IRS-4 and Ki-67 levels are significantly correlated with the presence of multifocal HCC. Moreover, upregulation of IRS-4 in HepG2 cells induced proliferation by a β-catenin/Rb/cyclin D mechanism, whereas downregulation of IRS-4 caused a loss in cellular polarity and in its adherence to collagen as well as a gain in migratory and invasive capacities, probably via an integrin α2 and focal adhesion cascade (FAK) mechanism.

## 1. Introduction

Hepatocellular carcinoma (HCC) is a globally prevalent liver cancer that has an incidence-to-mortality ratio near 1 [1]. Unfortunately, most cases of HCC are detected at late stages, which often leads to poor prognoses. Consequently, research efforts have been made to improve early identification and treatment for this deadly disease. Given that cancer is characterized by unregulated cellular proliferation, research investigating potential treatments emphasizes biochemical pathways involved in the cell cycle.

The insulin receptor substrate-4 (IRS-4) gene is on the X-chromosome [2] and belongs to the IRS family involved in the transmission of signals from the insulin and insulin-like growth factor-1 (IGF-1) receptors to downstream effectors in the liver [3]. Recently, the complete sequencing of 7416 [4] and 1220 [5] human cancer genomes revealed the deregulation of IRS-4 gene in cancer cells; furthermore, the data situate IRS-4 in a tumor growth-promoting role [4]. Other studies have found IRS-4 expression is significantly low in normal tissues [3,6]. In contrast, IRS-4 has been shown to be overexpressed in benign proliferative lesions such as uterine leiomyomas [7] and subungual exostosis [8], as well as in malignant diseases such as breast cancer [9], leukemia [10], lung cancer [4,11], and colorectal cancer [12,13].

In normal cells, IRS family proteins, such as IRS-1 and IRS-2, are widely expressed as they are necessary in insulin signaling processes [14]. IRS-1 and IRS-2 play important roles in the regulation of carbohydrate metabolism by insulin [14]. Upon receptor activation, IRS proteins are rapidly phosphorylated on tyrosine residues and then recruit downstream molecules that activate AKT and MAPK cascades leading to subsequent effector activation [14]. However, IRS-4 is able to constitutively activate AKT pathway because it lacks the ability to be inhibited by tyrosine phosphatase SHP-2 [15].

Moreover, IRS-4 has been reported to stimulate the ERK pathway in a PKC-dependent manner [16]. Dysregulation of AKT and ERK pathways has been implicated in the carcinogenesis of several human cancers. Despite the relations between IRS-4 and cell cycle dysregulation, the oncogenic mechanism of IRS-4 remains unclear. However, some data reveal that IRS-4 overexpression is associated with recurrent deletions in cis-regulatory elements in lung cancer [4]; similarly, chromosome translocations in T cells have been observed, which involves IRS-4 gene induction in acute lymphoblastic leukemia [10]. Recently, a novel epigenetic mechanism of IRS-4 upregulation has been described in melanoma [17]. The upregulation occurs via an increase of endogenous long intergenic non-protein-coding RNA-173, a natural sponge of microRNA-493 (miR-493), which in turn induces IRS-4 transcript degradation [18].

Previously, Cantarini et al. [19] showed the increase of mRNA levels of IRS-4 in 80% of the HCC samples analyzed. Moreover, our group has previously studied the role of IRS-4 in the proliferation of the rat liver after partial hepatectomy [3], as well as in the proliferation of the HepG2 cells stimulated by IGF-1 [16]. In sum, the data implicating IRS-4 in cancer cell proliferation in conjunction with the widespread prevalence of HCC led us to develop this pilot study, using both patient samples and experimental conditions in vitro, to unravel the possible role of IRS-4 in liver carcinogenesis.

## 2. Materials and Methods

### 2.1. Materials

Recombinant antibodies against IRS-4, αp85 were obtained from Upstate Biotechnology (Lake Placid, NY, USA). Antibodies against ERK 1/2, p-AKT (Thr 308), p-Rb (ser 807/811), Rb, E2F1, cyclin A, cyclin B, cyclin D1, cyclin E, cdk 2, cdk 4, p-cdk1, FAK, p-FAK (Tyr 925, Tyr 397), and β-tubulin were from Cell signaling Technology Inc. (Danvers, MA). Antibodies against p-ERK, p-AKT (Ser473), p-Tyr (PY99), and integrin α2 and β1 were acquired from Santa Cruz Biotechnology Inc (Santa Cruz, CA, USA). Antibodies against p-FAK (Tyr 407) were from BioSource Quality Controlled Biochemicals, Inc. (Morgan Hill, CA, USA). Goat anti anti-mouse IgG H&L chain specific peroxidase conjugate and anti-rabbit IgG conjugated to horseradish peroxidase were from Calbiochem (Barcelona, Spain). Phalloidin-FITC, PI3K inhibitor (Ly294002), p-FAK (Tyr 576, Tyr 861), and type I collagen were all from Sigma (St Louis, MO, USA). Antibodies against p-FAK (Tyr 577) were from Thermo Fisher Scientific (Waltham, MA, USA). All other reagents were of the highest grade of purity available.

### 2.2. Patients

Liver tissue from patients with HCC was acquired from surgical specimens at the time of surgery in the Hospital Universitario Gómez Ulla. The tumors were staged by the standard criteria using the American Joint Committee on Cancer (AJCC). All patients had given informed consent for the use of samples for research purposes, and this was approved by the Ethical Committee of our Institution. The study was carried out in accordance with the basic ethical principles of autonomy, beneficence, non-maleficence, and distributive justice, and its development followed the rules of Good Clinical Practice, the principles contained in the most recent Declaration of Helsinki (2013) and the Oviedo Convention (1997). The collected data and information complied with the current legislation on data protection (Organic Law 3/2018 December 5 on the Protection of Personal Data and the Guarantee of Digital Rights and Regulation (EU) 2016/679). The clinicopathological data, gender, and age of patients are summarized in (Supplementary Material, Table S1). The histological tumor grade was classified as well-differentiated (G1) or moderately differentiated (G2) carcinoma. Tumor samples were paraffin-embedded or frozen in liquid nitrogen immediately after removal and stored at −80 °C until use.

### 2.3. Cell Culture

The human hepatoblastoma cell line HepG2, HuH7, and Chang cells were obtained from ATCC and maintained in MEM (Gibco, Grand Island, NY, USA) or supplemented with 10% fetal bovine serum (FBS) and 1% antibiotic/anti-mycotic solution at 37 °C in a 5% CO2 humidified incubator. IRS-4 was overexpressed in HepG2 cells by transfection with pcDNA (IRS-4) or with the empty vector (pcDNA) as previously described [13]. IRS-4 silencing in HepG2 cells was performed in the conditions and with the oligos previously described [16].

To study the stimulatory effect of growth factors, HepG2 cells were starved for 72 h, then stimulated with IGF-1 (25 nM) for 30 min, or EGF (20 ng/mL) for 2, 5, and 10 min. Finally, cells were disrupted and lysed for further analyses as previously described [16].

### 2.4. Immunohistochemistry (IHC) and Immunocytochemistry (ICC)

In the IHC experiments, samples of liver tumors (T) were fixed in 4% paraformaldehyde and embedded in paraffin. The sections of 3 µm were incubated with rabbit polyclonal anti-IRS-4 (Upstate Biotechnology, Lake Placid, NY, USA), as described previously [2]. Immunostaining of the PCNA, Ki-67, and pH3 antigens in the histological sections was performed using routine techniques performed in the Pathology Department of the Hospital Central de la Defensa Gómez Ulla using the following antibodies PC10, SP6, and PHH3, respectively.

In the ICC experiments, HepG2 cells were cultured on glass coverslips and for some experiments were transfected as described above. Cells were fixed with 4% paraformaldehyde and permeabilized with 0.1% Triton X-100. Then, HepG2 cells were incubated with anti-IRS-4 (1/100), pERK1/2, and anti-integrin α2 (1/100) rabbit polyclonal antibodies, or antibody-free PBS as a nonspecific control. After incubation, cells were labeled using FITC-conjugated anti-rabbit Ig antibodies (1/1000 dilution) (Molecular Probes, Eugene, OR). Nuclei were stained by incubation with propidium iodide (Molecular Probes, Eugene, OR) and the HepG2 cells examined under a laser scanning confocal microscope (Leica TCS-SL). In another set of experiments, F-actin was labeled using Phalloidin-FITC.

### 2.5. Biochemical and Molecular Biology Methods

HepG2 cells and human liver biopsies were obtained for protein extraction, as previously described [16]. HepG2 cell lysates (40 μg of protein) or human liver homogenates (40 μg of protein) were analyzed by SDS-PAGE and western blot as previously described [16]. For immunoprecipitation (IP) experiments using HepG2 extracts, 250–500 µg of total protein was incubated overnight at 4 °C with 2 µg of specific anti-FAK or anti-IRS-4 antibodies. Next, protein G-agarose beads were added and the sample was incubated for 2 h at 4 °C. Negative controls (C-) were performed replacing the HepG2 sample with buffer and maintaining all other reagents used in the immunoprecipitation protocol. After washing three times with ice-cold lysis buffer, the immunocomplexes were analyzed by immunoblotting as previously described [16].

An MTT reduction assay was carried out according to the method described previously [20]. qPCR was performed as previously described [13] with minor modifications. Total RNA was isolated using RNeasy Mini Kit (Qiagen) in accordance with the manufacturer’s protocol. Contaminated genomic DNA was eliminated using RNase-free DNase (Qiagen). Total RNA (2 µg) was reverse transcribed into single-stranded cDNA using the AMV First Strand cDNA synthesis kit (Roche) in accordance with the manufacturer’s protocol. Real-time PCR amplification reactions were performed using the SYBR Green PCRMaster Mix (Applied Biosystems). The cycling conditions and the primers used to amplify IRS-1, IRS-4, and 18S have been previously described [13].

### 2.6. Cellular Adhesion to Collagen Experiments

Transfected or non-transfected, treated or untreated HepG2 cells were detached from culture dishes with trypsin/EDTA (Invitrogen, Barcelona, Spain). The reaction was then halted by 2-fold dilution with 1 mg/mL soybean trypsin inhibitor/PBS. Cell adhesion experiments were completed in 96-well plates coated with type I collagen (8 µg/cm^2^, 1 h at 37 °C). Furthermore, 3 × 104 cells were added to coated wells (100 µL aliquots) and incubated a 37 °C for different times. Non-adherent cells were carefully removed by washing them three times with PBS. Adherent cell density was determined using a MTT reduction assay. MTT stock solution in PBS buffer was added to the plate wells to obtain a final concentration of 0.5 mg/mL of MTT in media. Cells were incubated further for 2 h at 37 °C. Formazan crystals were dissolved in DMSO and absorbance was measured in a microplate reader. In some experiments, cells were incubated for 1 h at 37 °C with EGF (20 ng/mL) or with anti-integrin α2 or anti-integrin β1 antibodies.

### 2.7. Cell Migration Assay

Cell migration was assessed using Boyden chambers equipped with 8 µm porosity polyvinylpyrrolidone-free polycarbonate filters (Sigma, St Louis, MO, USA). Serum deprived HepG2 cells transfected with IRS-4 siRNA (R cells) or scrambled (S cells) oligos were washed, trypsinized, and resuspended in serum free medium containing 0.1% albumin, and 2 × 105 cells were placed in every upper chamber (200 µL aliquots). In some experiments, the cells were incubated in the presence of Ly294002 (20 µM). The lower chamber was filled with MEM containing 0.1% BSA with or without EGF (50 ng/mL) and diluted type I collagen (50 µg/mL) was used as a chemotactic factor. The Boyden chamber was incubated for 24 h at 37 °C to allow possible cell migration through the membrane into the bottom chamber. Membranes were stained with hematoxylin (Sigma, St Louis, MO, USA) for 5 min and migration was quantified by counting the cells that had migrated to the lower surface of the polycarbonate filters using a Nikon microscope with bright-field optics (×40 magnification). For each filter, six randomly chosen microscopic fields were photographed and the number of cells in each field counted and averaged (mean ± SD). Results are expressed as number of migrated cells per field.

### 2.8. Statistical Analysis

Statistical differences among the clinical parameters studied were performed either by ANOVA analyses or by Student’s t-tests, depending on the number of groups compared. The correlations between percentage of stained nuclei with IRS-4 and the other biomarkers (PCNA, Ki-67, or pH3) were analyzed using Pearson’s correlation coefficient (r). The possible relation between the levels of IRS-4 + Ki-67 and the clinical outcome was evaluated using the Odds ratio (OR), considering the control group as patients who had IRS-4 + Ki- 67 ≥ 70 and the exposed group as those who had IRS-4 + Ki-67 < 69. Statistical differences in the experiments carried out with HepG2 cells were analyzed by ANOVA analyses or by Student’s t tests as a function of the number of groups compared. At least three independent experiments were performed to obtain each result. The levels of significance were set at *p* < 0.05 (*), *p* < 0.01 (**), and *p* < 0.001 (***).

## 3. Results

### 3.1. Localization of IRS-4 in Human HCC

**Figure 1:**
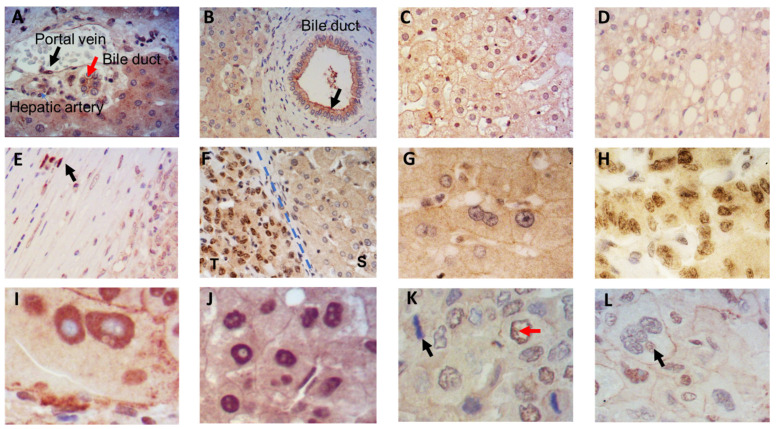
Immunohistochemical staining of IRS-4 in surrounding tissue of the tumoral region (**A**–**E**) and in the tumors (**F**–**L**) of human HCC. Paraffin embedded liver biopsies were stained with anti-IRS-4 antibody. T = tumoral region; S = surrounding tissue (A–E 20× magnification and F–L 40× magnification).

IRS-4 expression was evaluated using immunohistochemistry on liver biopsies of 31 patients with HCC. Supplementary Material Table S1 provides a detailed description of the study sample. IRS-4 expression was also assessed in two samples of healthy tissue adjacent to the tumor using immunoblot techniques. Ultimately, the expression of IRS-4 was analyzed in three regions: tumoral cells, fibrous connective capsules, and normal surrounding hepatocytes. The cells and structures beyond the connective capsule had normal characteristics. We used this region to study the expression of IRS-4 in liver parenchyma, portal triads, and canaliculi (Figure 1). In the portal triads, IRS-4 was observed in the nuclei of portal vein endothelial cells (black arrow) and in the membranes of the bile duct epithelial cells (red arrow) (Figure 1A). IRS-4 expression is evident in both the cytosol and the apical membranes in epithelial cells of canaliculi (Figure 1B). In the healthy tissue, hepatocytes were observed to be arranged in plates, with IRS-4 expression primarily localized in the membrane and cytosol (Figure 1C). In liver parenchyma affected by steatosis, IRS-4 was also observed to be localized in the membrane and cytosol (Figure 1D). In the connective tissue of the portal area, IRS-4 was present in the nuclei of a few fibroblasts (Figure 1E, black arrow). Interestingly, when compared to healthy surrounding tissue (S), tumoral tissue (T) exhibited a dramatic elevation in nuclear IRS-4 (Figure 1F), which in the vast majority of the biopsies studied delineated a clear border between tumoral tissue and healthy tissue.

When observed at a higher magnification, it became apparent that IRS-4 was absent in the nuclei of healthy hepatocytes from surrounding non-tumoral tissue (Figure 1G), whereas nuclear IRS-4 was present in a high proportion of tumoral cells (Figure 1H). Nuclear localization of IRS-4 was also found in giant cells (Figure 1I), as well as in well-differentiated hepatocytes from tumoral tissue (Figure 1J). However, IRS-4 was not associated to chromosomes in metaphase (Figure 1K, black arrow), nor observed to be localized in the nucleoli (Figure 1I,J). The staining of tumoral hepatocytes from HCC revealed that IRS-4 was located in the nucleus with two clear patterns. First, in well-differentiated tumoral cells, IRS-4 localization was homogenously disseminated within the nucleus with exception of the nucleolus. Second, in tumoral cells, IRS-4 was present in the internal region of the nuclear membrane (Figure 1K, red arrow). IRS-4 expression was not observed in some aberrant cells with giant nuclei (Figure 1L, black arrow). Furthermore, several “owl eye” hepatocytes expressing nuclear IRS-4 in the tumoral tissue were detected.

**Figure 2:**
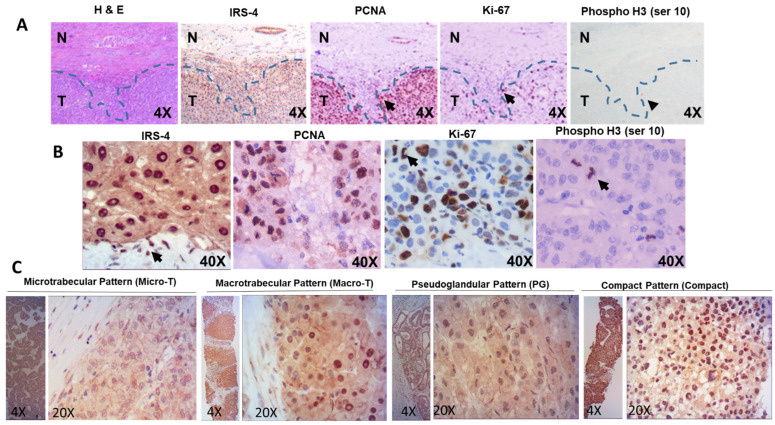
Immunohistochemical staining of IRS-4, PCNA, Ki-67, and pH3 (**A**,**B**). Immunohistochemical staining of IRS-4 in different architectural patterns of HCC (**C**). H&E = hematoxylin and eosin staining.

PCNA, Ki-67, and pH3 are well accepted cancer biomarkers, thus, we decided to compare IRS-4 expression pattern with those of the three proteins using immunohistochemistry methods. Representative results are shown in Figure 2. We observed that PCNA was specifically localized in the nuclei of HCC tumoral cells and its levels decreased dramatically in the fibrous connective capsule. However, the IRS-4 expression profile differed from that of PCNA; IRS-4 was also found in the fibrous connective capsule, likely in immune cells, while PCNA was not. Ki-67 was also observed in the nuclei of tumoral cells but in lower levels compared to that of IRS-4 and PCNA. The final biomarker analyzed, pH3, was scarcely detected and only in cells in metaphase (Figure 2A,B). We found nuclear expression of IRS-4 in four architectural patterns of HCC: microtrabecular, macrotrabecular, glandular, and compact patterns (Figure 2C). Preliminary results show that cells with nuclear IRS-4 were more abundant in the compact pattern (Figure 2C).

**Figure 3:**
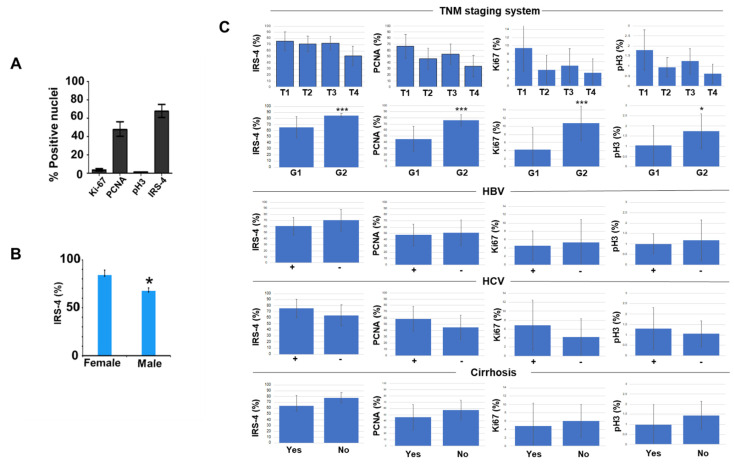
Bar graphs representing quantitative analysis of immunohistochemical staining. (**A**). Percentage of positive nuclei of IRS-4, PCNA, Ki-67, and pH3. (**B**). Percentage of IRS-4 positive nuclei in female and male samples. (**C**). Percentage of positive nuclei of IRS-4, PCNA, Ki-67, and pH3 compared with notable patient measures. Data are represented as mean and SD. Significance levels using student t test were: * *p* < 0.05; *** *p* < 0.001.

The percentage of tumor cells with nuclear IRS-4 was 69.9 ± 3.2%, followed by nuclear PCNA 52.1 ± 7.8%, nuclear Ki-67 6.1 ± 1.1% (Figure 3A), and finally pH3 with only 1.3 ± 0.2% (Figure 3A). We observed that the percentage of tumoral cells with nuclear IRS-4 was higher in women than in men (Figure 3B).

We did not observe significant differences in the IRS-4, PCNA, Ki-67, and pH3 values when stratifying the patients according to the T value (TNM staging system) (Figure 3C). Infection by virus B (HBV) or C (HCV) or the presence of cirrhosis was not associated with significant changes in IRS-4 levels (Figure 3C). Interestingly, the markers studied (IRS-4, PCNA, Ki-67, or pH3) increased significantly in the group G2 with respect to G1 group (Figure 3C), which suggests a relation with tumor grade.

**Figure 4:**
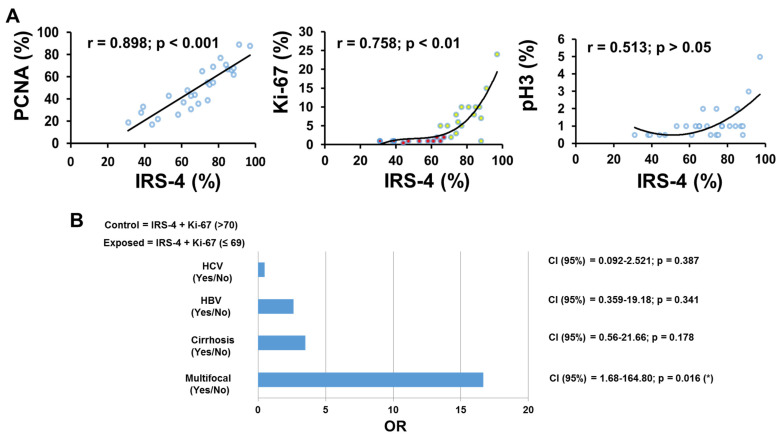
Relation between the percentage of IRS-4 compared with percentage of PCNA, Ki-67, and pH3 (**A**). Percentage of IRS-4 expression as a function of PCNA, Ki-67, and pH3. (**B**). Odds ratio of P1 (exposed-red points) and P2 (control-yellow points) groups as defined in the Results section *p* < 0.05 (*).

To establish possible relations among the above mentioned immunohistochemical parameters, we estimated the Pearson’s correlation coefficient among them in the group of HCC liver samples (Figure 4A). Interestingly, we observed a strong correlation between nuclear IRS-4 and nuclear PCNA, and between nuclear IRS-4 and nuclear Ki-67; however, we did not find a significant correlation between nuclear IRS-4 and pH3.

**Figure 5:**
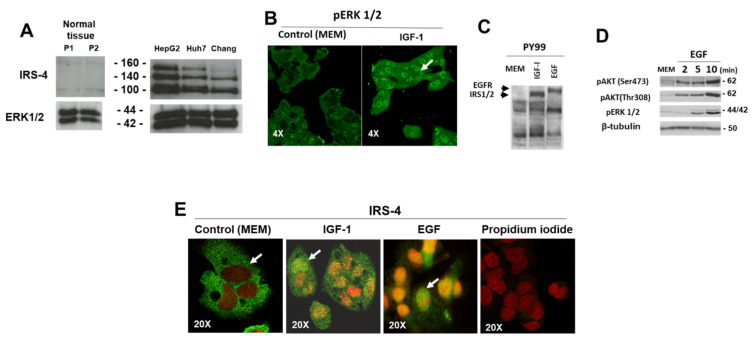
Study of IRS-4 and cell cycle-related proteins using immunostaining and immunoblotting techniques. (**A**). Western blot analysis of IRS-4 expression in normal liver biopsies of two patients and in HepG2, Huh7, and Chang cells. ERK 1/2 was used as loading control. This experiment is representative of three others with similar results. (**B)**. HepG2 cells treated with IGF-1 and stained with anti-pERK1/2 antibody (green). (**C**). Western blot analysis of tyrosine phosphorylation following either IGF-1 or EGF stimulation. (**D**). Time course studies of phosphorylation after EGF stimulation. (**E**). HepG2 cells stained with anti-IRS-4 antibody (green) and with propidium iodine (red). For negative control, the anti-IRS-4 antibody was omitted and was incubated only with propidium iodine. The experiments shown in panels (**B**–**E**) are representative of three–five others with similar results.

However, we noticed a significant curvilinear correlation between IRS-4 and Ki-67, with two slopes, which allowed us to define two populations: P1-red points (IRS-4 + Ki-67 ≤ 69) and P2-yellow points (IRS-4 + Ki-67 > 70%) (Figure 4A). Interestingly, when we compare the frequency in the multifocal phenotype between both groups, we observe a much higher proportion in P1 with respect to P2, with an OR = 16.66 (CI (95%) = 1.68–164.80; P = 0.016) (Figure 4B). Additionally, we compared IRS-4 expression by immunoblot in two normal human liver samples, from tissue adjacent to the tumor, with the corresponding results of IRS-4 levels in HepG2, HuH 7, and Chang cells (Figure 5A). In HepG2 cells, we observed the presence of three proteins as previously described [16]. We observed low levels of IRS-4 in normal liver tissue compared with that of HCC cell lines (Figure 5A). In order to further evaluate a potential mechanism of IRS-4 action in liver cancer cells, we performed in vitro studies using HepG2 cells transfected with either the IRS-4 gene or with siRNA.

### 3.2. Role of IRS-4 in the Proliferation of HepG2 Cells

Previously, we assessed the functionality of IGF-1 and EGF receptors in HepG2 cells. HepG2 cells were incubated with IGF-1 (25 nM) for 30 min, after which the location of *p*-ERK1/2 inside the cell was studied. We can observe the increase of the active ERK kinase in the nucleus of cells treated with IGF-1 (Figure 5B). Using a PY99 antibody, we observed the tyrosine phosphorylation of several proteins after IGF-1 (25 nM) or EGF (20 ng/mL) stimulation of HepG2 cells for 30 min. The patterns of Tyr phosphorylation of both growth factors were different—IGF-1 incubation induced the phosphorylation of a 180 kDa band, which could correspond to IRS-1/2, whereas the EGF incubation increased the phosphorylation of an upper band compatible with EGF receptor protein (Figure 5C). Time course studies using EGF showed a rapid increase of pAKT (Ser473 and Thr308) and pERK1/2 (Figure 5D), confirming the expression of both types of functional receptors in HepG2 cells.

To correlate the observations obtained in HCC patients with those obtained in HepG2, we studied the subcellular localization of IRS-4 in HepG2 cells using immunofluorescence microscopy in basal conditions (in absence of FBS during 72 h) and after incubation with IGF-1 (25 nM) or with EGF (20 ng/mL) for 30 min (Figure 5E). IRS-4 (green) was found in the cytosol of serum-starved cells (Figure 5E) and after IGF-1 or EGF stimulation, IRS-4 localized in the nuclei. Propidium iodine was used as a nuclear stain (red).

**Figure 6:**
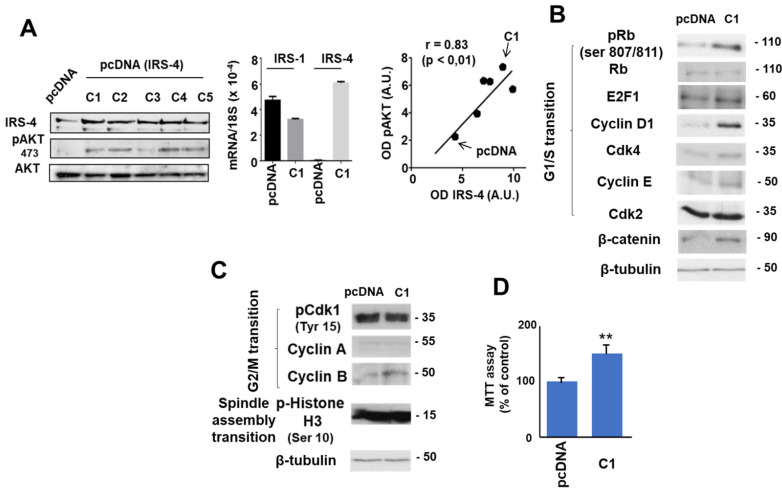
Immunoblot analyses of IRS-4 and other cell cycle regulatory proteins. (**A**). Western blot demonstrating IRS-4 pcDNA transfection of five cell lines (C1–C5). (**B**). Western blot of proteins involved in regulatory signaling pathways corresponding to G1/S transition in C1 and pcDNA colonies. (**C**). Western blot of proteins involved in regulatory signaling pathways corresponding to G2/M and spindle assembly transitions. (**D**). Cell proliferation compared to IRS-4 levels of C1 and pcDNA colonies. This experiment is the mean ± ESM of four performed in triplicate. The western blot experiments are representative of three–four others with similar results. ** *p* < 0.01.

To study the effect of overexpression of IRS-4 in proliferation of HepG2 cells, we stably transfected HepG2 cells with pcDNA (IRS-4) in five lines (C1-C5). The corresponding results are shown in (Figure 6A). Our observations demonstrate that pcDNA (IRS-4) transfection increased IRS-4 protein levels in the five lines studied (C1-C5) in comparison to the control group (pcDNA) (Figure 6A; left panel). This effect was specific for IRS-4 with a slight effect on IRS-1 levels, measured by qPCR (Figure 6A; middle panel). Moreover, we studied pAKT (Ser473) activation in all transfected cells and observed a strong correlation between IRS-4 and pAKT (ser 473) levels as measured by western blot (r = 0.83, *p* ˂ 0.01) (Figure 6A; right panel).

We continued this study with the C1 colony because it expressed the highest levels of IRS-4. Overexpression of IRS-4 was accompanied by the increase of several proteins involved in the regulation of G1/S transition of the cell cycle, mainly in the amounts of cyclin D1 and β-catenin as well in the phosphorylation of Rb (ser 807/811) (Figure 6B). Total concentrations of Rb and E2F1 (the main target of Rb), CDK4, CDK2, and cyclin E did not change substantially in IRS-4-overexpressing cells. Moreover, we observed an increase in cyclin B without changes in pCDK1(tyr15) or in cyclin A, which are involved in the control of G2/M transition of cell cycle (Figure 6C). Furthermore, no changes in pH3(ser 10), a protein essential in the spindle assembly transition, were observed following IRS-4 overexpression (Figure 6C). Ultimately, elevated IRS-4 levels were accompanied by increased proliferation with respect to control cells after three days of culture in the presence of 10% FBS (Figure 6D).

### 3.3. Study of the Role of IRS-4 Depletion on Cell Morphology, Integrin α2 Distribution, Cell Adhesion, and Migration of HepG2 Cells

**Figure 7:**
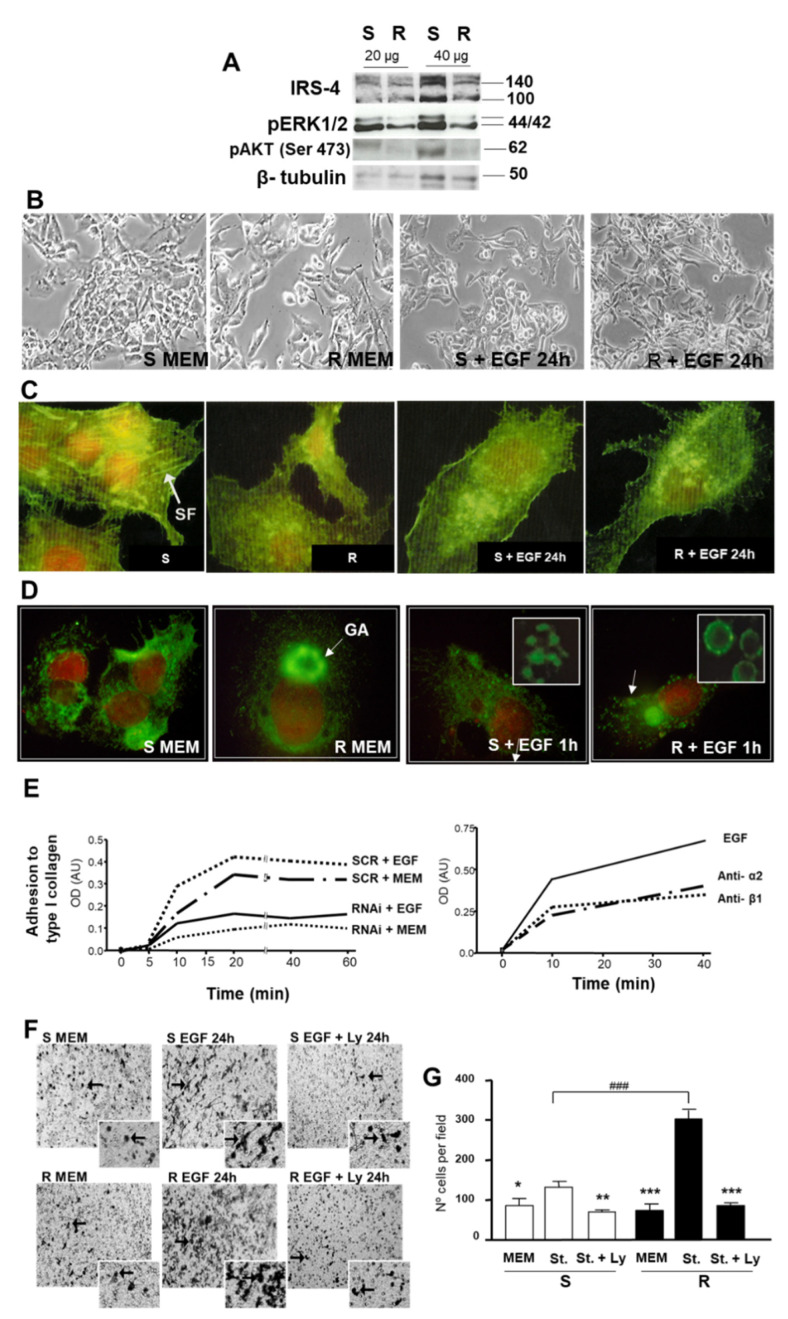
Microscopic and immunoblot analyses of cellular changes in morphology, adhesion, and invasive capacity. (**A**). Western blot assessing the expression and phosphorylation of AKT and ERK 1/2 in control (S) compared with IRS-4 knockdown (R) cells. (**B**). Microscopic images of demonstrating changes in cell morphology in (R) cells compared to (S) cells in basal conditions and after 24 h of EGF incubation. (**C**). Confocal fluorescent microscopic images of (S) and (R) cells stained with Phalloidin-FITC in basal conditions and after 24 h of EGF incubation. (**D**). Confocal fluorescent microscopic images of (S) and (R) cells stained with anti-integrin α2 (green) in basal conditions and after 1 h of EGF incubation. (**E**) Graphical representation of HepG2 cell adhesion to type 1 collagen. These experiments are representative of three others with similar results. (**F**). Microscopic images of cell migration assessed using a Boyden chamber in basal conditions, 24 h of EGF + type I collagen (St.) incubation, and 24 h St. + Ly294002 incubation. (**G**). Bar graph representation of (F). For comparisons between St. and the other groups, the significance levels using ANOVA test were: * *p* < 0.05; ** *p* < 0.01; *** *p* < 0.001. For comparisons between (R) and (S) cells the significance levels using ANOVA test was: ### *p* < 0.001. St. = stimulating conditions (EGF + type I collagen).

The decrease in IRS-4 levels by interference technology in R cells produced a simultaneous reduction in cell number with respect to control cell (S) as previously described [16]. The decrease in IRS-4 (R) levels correlated with the decrease in pERK 1/2 and pAKT (Ser473), when compared with control cells (S) (Figure 7A). Changes in cell morphology, including elongation with the emission of irregular prolongations, as well as a greater separation between cells in R with respect to S cells, was also observed (Figure 7B). These changes became more evident when EGF (20 ng/mL) was added to the culture medium of R cells for 24 h (Figure 7B). The study of the cells stained with Phalloidin-FITC and assessed by confocal fluorescent microscopy revealed important changes in the stress fibers (SF). SF were more abundant in S (control cells) than in R (IRS-4 depleted cells). We also observed that EGF (20 ng/mL) treatment for 24 h produced a profound impact in the number of SF, which was similar in S and in R cells (Figure 7C).

The effect of IRS-4 depletion on cellular distribution of integrin α2 (green) was assessed using confocal microscopy. Figure 7D demonstrates a clear difference in the intracellular localization of integrin α2 in R and S cells in both basal conditions (in the presence of MEM) or after 1 h of EGF (20 ng/mL) stimulation. Notably, increases in the accumulation of integrin α2 in the Golgi apparatus as well as in intracellular vesicles in R cells with respect to S cells were recorded (Figure 7D, inset). The nuclei were counterstained with propidium iodide (red). These data suggest changes in the intracellular traffic of integrin α2 in IRS-4 depleted cells. Since IRS-4 knockdown could alter integrin α2 distribution, we went on to explore the effect of IRS-4 depletion on cell adhesion to type I collagen. Figure 7D (left panel) illustrates that when IRS-4 was reduced, cell adhesion to collagen diminished regardless of presence or absence of EGF (20 ng/mL). To check whether cell adhesion was mediated by integrin α2 and β1, we performed cell adhesion experiments in non-transfected cells using integrin-blocking antibodies. Pretreatment of HepG2 cells was done with anti-integrin α2 or anti-integrin β1 inhibit cell adhesion (Figure 7D; right panel).

Next, cell migration was measured in the Boyden chamber. In IRS-4-depleted cells (R), migration in the presence of chemotactic factors (EGF; 50 ng/mL and type I collagen, 50 µg/mL) was greater than that observed in control cells (S). This effect was significantly inhibited by Ly294002, a PI3K inhibitor (Figure 7F). Figure 7G shows the average of the experiments performed.

**Figure 8:**
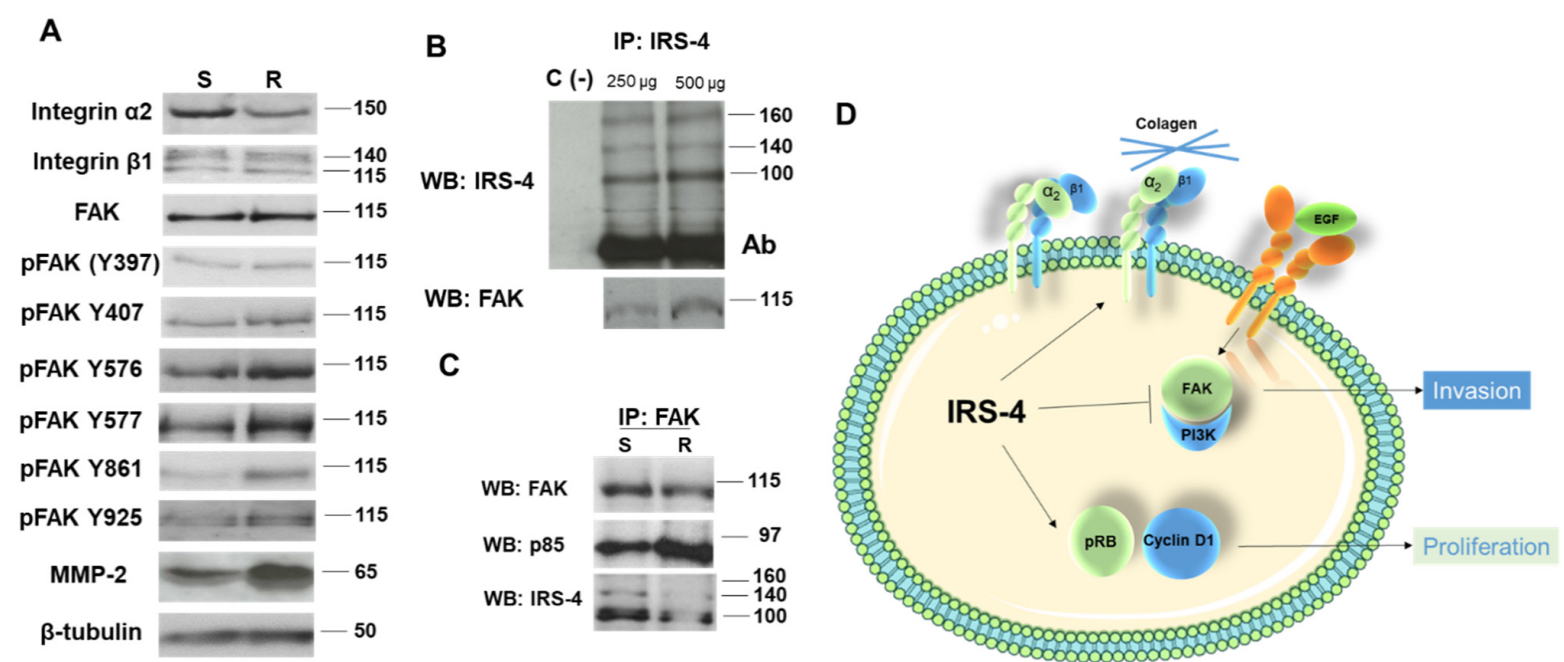
Immunoblot analyses of proteins involved in cellular adhesion and the FAK-p85PI3K complex. (**A**). Western blot of adhesion proteins and FAK phosphorylation at various residues in control (S) and IRS-4 knockdown (R) cells. (**B**). Reciprocal western blot/IP of FAK and IRS-4. (**C**). Reciprocal western blot/IP of FAK and p85-PI3K with IRS-4 western blot. (**D**). Visual representation of proposed IRS-4 mechanism of action in proliferation/invasion.

The changes in the distribution of integrin α2 observed in microscopic experiments were corroborated by immunoblot techniques. IRS-4 knockdown decreased integrin α2 levels but not integrin β1 and FAK levels (Figure 8A). However, the phosphorylation of several tyrosine residues (positions 576, 577, 861 and 925) increased in R cells with respect to S cells (control conditions) (Figure 8A). In Figure 8A, we also show the levels of MMP-2 in R and S cells, as it can be seen that the decrease in IRS-4 levels favors the increase of MMP-2.

Next, we show the physical interaction between IRS-4 and FAK by reciprocal immunoprecipitation (IP) in Figure 8B,C. We observed the increase of the IRS-4-FAK complex immunoprecipitated with the amount of protein (250 or 500 µg) of the input lysate (Figure 8B).

When evaluating the impact of an IRS-4 knockdown on the association between FAK and p85-PI3K, we observed that the decrease in IRS-4 caused an increase in the association between FAK and p85-PI3K (Figure 8C).

## 4. Discussion

The present paper demonstrates for the first time the overexpression of nuclear IRS-4 in human HCC and its involvement in liver cancer cell proliferation as well in collagen cell adhesion and cell motility. Previously, Cantarini et al. [19] showed an increase of IRS-4 mRNA levels in 80% of the HCC samples analyzed. Moreover, our group has shown the role of IRS-4/PI3K cascade in the regeneration of rat liver [3]. Present results suggest that nuclear IRS-4 expression could be an interesting biomarker of HCC because it is correlated with classical markers of carcinogenesis (PCNA and Ki-67). Curiously, low levels of nuclear IRS-4 and Ki-67 were related to having a greater probability of a multifocal HCC phenotype. There are some molecules, such as cyclin D1 and Ki-67, that activate the cell cycle and could be inversely related with the invasiveness of certain type of tumors.

Several studies have shown the arrest of cell proliferation in the invasion front of colonic carcinomas characterized by low levels of cyclin D1, β-catenin, and Ki-67 [21,22,23]. In fact, the cyclin D1 oncogene is highly expressed in many cancers and, despite its proliferation-activating properties, it has been linked to a less malignant phenotype [24]. According to these observations, hepatocellular carcinoma cells invade during the G1 phase of the cell cycle [25]. Thus, it appears that epithelial mesenchymal transition (EMT)-like behavior from multiple cancer types may be linked to G1/G0 cell cycle arrest [26]. IRS-4 could be involved in this process, favoring tumor proliferation in certain areas and decreasing the invasive capacity on the invasive front of the tumor, which could explain the phenomenon of patients with a lower proportion of IRS-4 and Ki-67 having a greater number of foci.

In the IRS-4 knockdown HepG2 cell experiments, we observed a decrease of stress fiber formation and cell adhesion due to integrin α2 downregulation as well as an increase in chemotactic induced cell motility. These data provide evidence for the inhibitory effect IRS-4 exerts on EMT. Moreover, we observed an increase of metalloproteinase 2 (MMP-2) production in IRS-4 depleted cells. A very recent study shows that decreases in IRS-4 levels causes a reduction of E-cadherin, another sign of EMT activation [11]. Moreover, IRS-4-depleted cells responded more efficiently than control cells to the chemotactic stimuli (collagen type I and EGF). This effect was completely blocked by Ly294002 (a PI3K inhibitor), so we focused on the FAK-PI3K complex when evaluating cell motility.

Currently, the precise molecular mechanism by which IRS-4 affects the FAK-PI3K complex is unknown. Preliminary results suggest that IRS-4 could destabilize the association between FAK and p85-PI3K. Similarly, previous studies show that IRS-4 binds and activates Slingshot-1 (SSH-1) phosphatase and promotes cofilin dephosphorylation, an essential process in cell migration [27]. Moreover, IRS-4 depletion appeared to activate FAK phosphorylation in several tyrosine residues (576, 577, 861, and 925) that are necessary in the acquisition of an invasive cellular phenotype [28].

This pilot study, in conjunction with recent research, presents IRS-4 as a notable protein implicated in the development of cancer. In two independent genome-wide association studies (GWAS) using DNA from 7416 and 1220 donors of different cancer types, IRS-4 has been identified as one of the best candidates involved in the promotion of carcinogenesis [4,5]. Evidently, the role of IRS-4 in cellular processes implicated in carcinogenesis is a noteworthy avenue for investigation. In the present study, we observed a bimodal effect of IRS-4, both in the proliferation and invasiveness of liver cancer (Figure 8D). In vitro, we observed translocation of IRS-4 to the nucleus after incubation of HepG2 cells, which could be involved in IGF-1-stimulated proliferation [16]. Additionally, IRS-4 overexpression in HepG2 cells leads to AKT activation, GSK3 phosphorylation, and an increase in β-catenin/cyclinD1 levels, which are necessary to complete the G1/S transition. Similar results have been observed following overexpression of IRS-4 in RKO, a colon cancer cell line [13]. To date, our results suggest that IRS-4 behaves like cyclin D1 and Ki-67, both of which are robust markers of tumor proliferation. However, for tumoral cells to gain an invasive capacity, cell cycle arrest is required, which depends on the decrease of cyclin D1 and Ki-67 in the front of tumor [21,22,23]. Our findings suggest that IRS-4′s role in the process of a tumoral cell gaining invasive capacity is akin to those of the proteins discussed above.

## 5. Conclusions

Collectively, our results suggest that the overexpression of IRS-4 may increase liver cancer growth, but its reduction together with Ki-67 could be important in the development in the multifocal phenotype.

## Data Availability

The data used to support the findings of the present study are available from the corresponding author upon request.

## Supplementary Materials

The following are available online at https://www.mdpi.com/article/10.3390/cancers13112560/s1, Table S1: Clinical characteristics of the study population and autoradiographs of western blot and antibodies are shown in the supplementary materials.
