# Supplementary material for: Possible Role of IRS-4 in the Origin of Multifocal Hepatocellular Carcinoma
Source: Cancers (Basel). 2021 May 23;13(11):2560. doi: 10.3390/cancers13112560 (PMC8197110; doi:10.3390/cancers13112560)
Supplement: Supplementary file 1 [file cancers-13-02560-s001.zip › cancers-1225413-supplementary.pptx]

## Slide 1
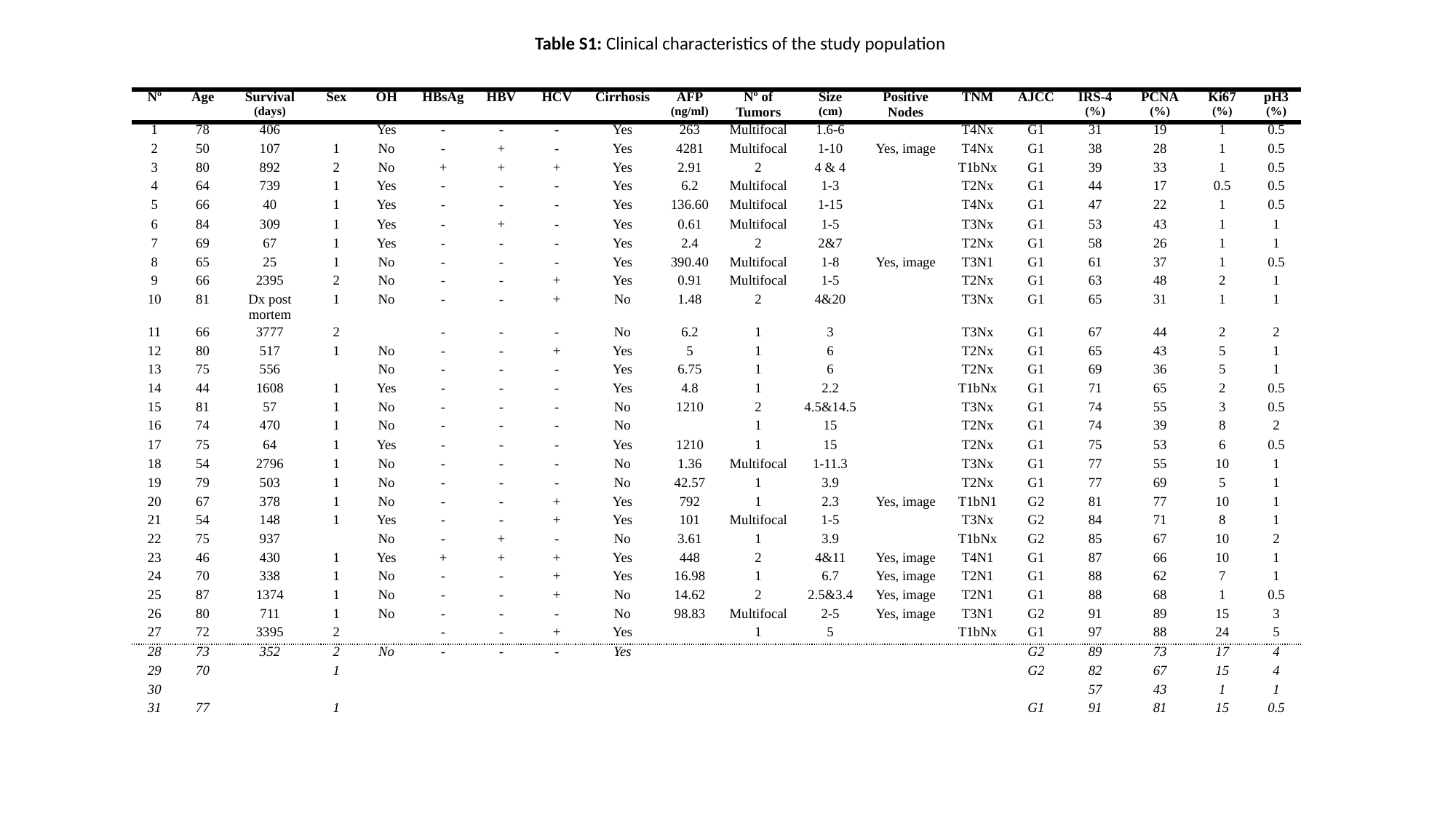

Table S1: Clinical characteristics of the study population
| Nº | Age | Survival (days) | Sex | OH | HBsAg | HBV | HCV | Cirrhosis | AFP (ng/ml) | Nº of Tumors | Size (cm) | Positive Nodes | TNM | AJCC | IRS-4 (%) | PCNA (%) | Ki67 (%) | pH3 (%) |
| --- | --- | --- | --- | --- | --- | --- | --- | --- | --- | --- | --- | --- | --- | --- | --- | --- | --- | --- |
| 1 | 78 | 406 | | Yes | - | - | - | Yes | 263 | Multifocal | 1.6-6 | | T4Nx | G1 | 31 | 19 | 1 | 0.5 |
| 2 | 50 | 107 | 1 | No | - | + | - | Yes | 4281 | Multifocal | 1-10 | Yes, image | T4Nx | G1 | 38 | 28 | 1 | 0.5 |
| 3 | 80 | 892 | 2 | No | + | + | + | Yes | 2.91 | 2 | 4 & 4 | | T1bNx | G1 | 39 | 33 | 1 | 0.5 |
| 4 | 64 | 739 | 1 | Yes | - | - | - | Yes | 6.2 | Multifocal | 1-3 | | T2Nx | G1 | 44 | 17 | 0.5 | 0.5 |
| 5 | 66 | 40 | 1 | Yes | - | - | - | Yes | 136.60 | Multifocal | 1-15 | | T4Nx | G1 | 47 | 22 | 1 | 0.5 |
| 6 | 84 | 309 | 1 | Yes | - | + | - | Yes | 0.61 | Multifocal | 1-5 | | T3Nx | G1 | 53 | 43 | 1 | 1 |
| 7 | 69 | 67 | 1 | Yes | - | - | - | Yes | 2.4 | 2 | 2&7 | | T2Nx | G1 | 58 | 26 | 1 | 1 |
| 8 | 65 | 25 | 1 | No | - | - | - | Yes | 390.40 | Multifocal | 1-8 | Yes, image | T3N1 | G1 | 61 | 37 | 1 | 0.5 |
| 9 | 66 | 2395 | 2 | No | - | - | + | Yes | 0.91 | Multifocal | 1-5 | | T2Nx | G1 | 63 | 48 | 2 | 1 |
| 10 | 81 | Dx post mortem | 1 | No | - | - | + | No | 1.48 | 2 | 4&20 | | T3Nx | G1 | 65 | 31 | 1 | 1 |
| 11 | 66 | 3777 | 2 | | - | - | - | No | 6.2 | 1 | 3 | | T3Nx | G1 | 67 | 44 | 2 | 2 |
| 12 | 80 | 517 | 1 | No | - | - | + | Yes | 5 | 1 | 6 | | T2Nx | G1 | 65 | 43 | 5 | 1 |
| 13 | 75 | 556 | | No | - | - | - | Yes | 6.75 | 1 | 6 | | T2Nx | G1 | 69 | 36 | 5 | 1 |
| 14 | 44 | 1608 | 1 | Yes | - | - | - | Yes | 4.8 | 1 | 2.2 | | T1bNx | G1 | 71 | 65 | 2 | 0.5 |
| 15 | 81 | 57 | 1 | No | - | - | - | No | 1210 | 2 | 4.5&14.5 | | T3Nx | G1 | 74 | 55 | 3 | 0.5 |
| 16 | 74 | 470 | 1 | No | - | - | - | No | | 1 | 15 | | T2Nx | G1 | 74 | 39 | 8 | 2 |
| 17 | 75 | 64 | 1 | Yes | - | - | - | Yes | 1210 | 1 | 15 | | T2Nx | G1 | 75 | 53 | 6 | 0.5 |
| 18 | 54 | 2796 | 1 | No | - | - | - | No | 1.36 | Multifocal | 1-11.3 | | T3Nx | G1 | 77 | 55 | 10 | 1 |
| 19 | 79 | 503 | 1 | No | - | - | - | No | 42.57 | 1 | 3.9 | | T2Nx | G1 | 77 | 69 | 5 | 1 |
| 20 | 67 | 378 | 1 | No | - | - | + | Yes | 792 | 1 | 2.3 | Yes, image | T1bN1 | G2 | 81 | 77 | 10 | 1 |
| 21 | 54 | 148 | 1 | Yes | - | - | + | Yes | 101 | Multifocal | 1-5 | | T3Nx | G2 | 84 | 71 | 8 | 1 |
| 22 | 75 | 937 | | No | - | + | - | No | 3.61 | 1 | 3.9 | | T1bNx | G2 | 85 | 67 | 10 | 2 |
| 23 | 46 | 430 | 1 | Yes | + | + | + | Yes | 448 | 2 | 4&11 | Yes, image | T4N1 | G1 | 87 | 66 | 10 | 1 |
| 24 | 70 | 338 | 1 | No | - | - | + | Yes | 16.98 | 1 | 6.7 | Yes, image | T2N1 | G1 | 88 | 62 | 7 | 1 |
| 25 | 87 | 1374 | 1 | No | - | - | + | No | 14.62 | 2 | 2.5&3.4 | Yes, image | T2N1 | G1 | 88 | 68 | 1 | 0.5 |
| 26 | 80 | 711 | 1 | No | - | - | - | No | 98.83 | Multifocal | 2-5 | Yes, image | T3N1 | G2 | 91 | 89 | 15 | 3 |
| 27 | 72 | 3395 | 2 | | - | - | + | Yes | | 1 | 5 | | T1bNx | G1 | 97 | 88 | 24 | 5 |
| 28 | 73 | 352 | 2 | No | - | - | - | Yes | | | | | | G2 | 89 | 73 | 17 | 4 |
| 29 | 70 | | 1 | | | | | | | | | | | G2 | 82 | 67 | 15 | 4 |
| 30 | | | | | | | | | | | | | | | 57 | 43 | 1 | 1 |
| 31 | 77 | | 1 | | | | | | | | | | | G1 | 91 | 81 | 15 | 0.5 |

## Slide 2
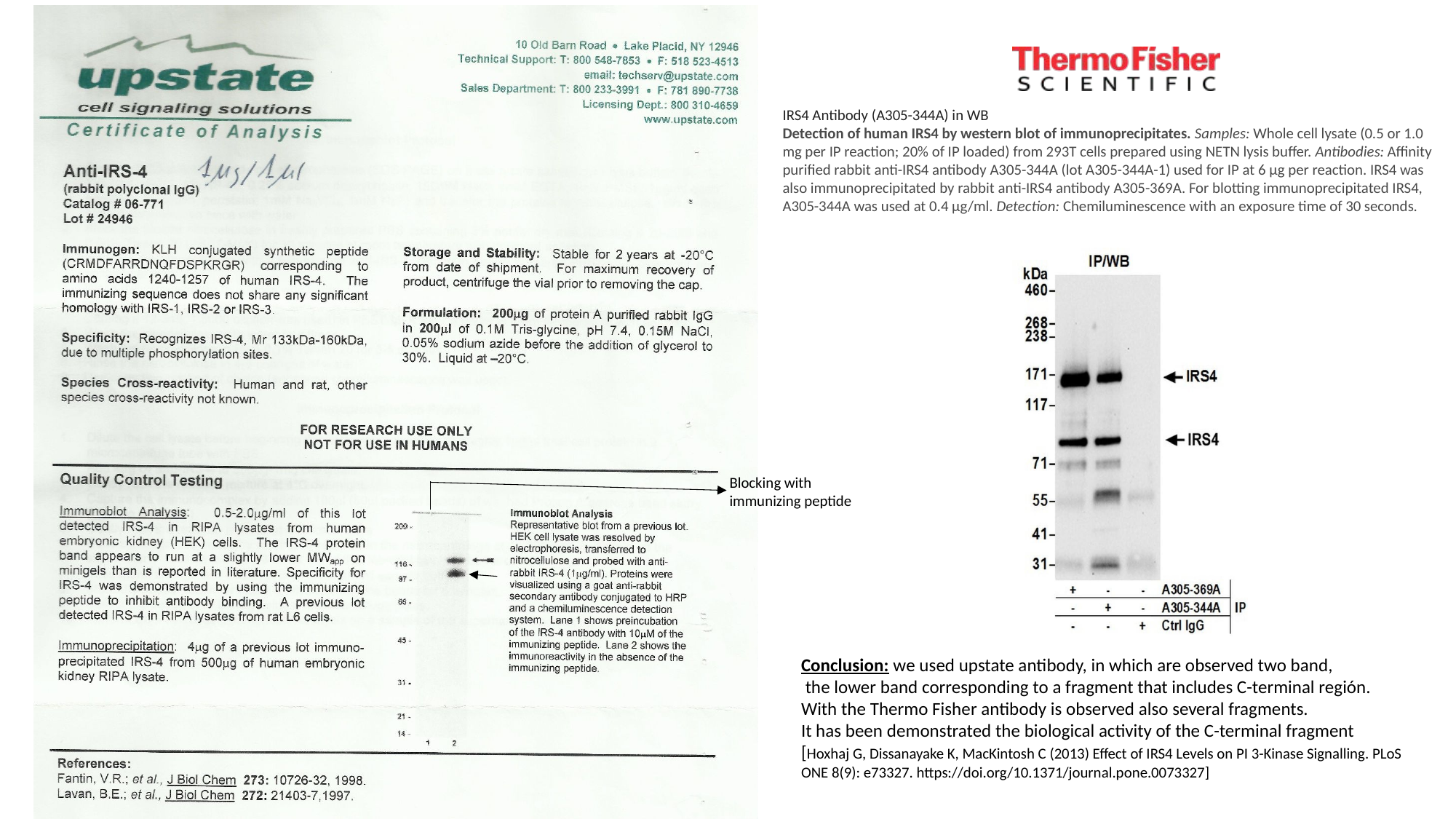

IRS4 Antibody (A305-344A) in WB
Detection of human IRS4 by western blot of immunoprecipitates. Samples: Whole cell lysate (0.5 or 1.0 mg per IP reaction; 20% of IP loaded) from 293T cells prepared using NETN lysis buffer. Antibodies: Affinity purified rabbit anti-IRS4 antibody A305-344A (lot A305-344A-1) used for IP at 6 µg per reaction. IRS4 was also immunoprecipitated by rabbit anti-IRS4 antibody A305-369A. For blotting immunoprecipitated IRS4, A305-344A was used at 0.4 µg/ml. Detection: Chemiluminescence with an exposure time of 30 seconds.
Blocking with
immunizing peptide
Conclusion: we used upstate antibody, in which are observed two band,
 the lower band corresponding to a fragment that includes C-terminal región.
With the Thermo Fisher antibody is observed also several fragments.
It has been demonstrated the biological activity of the C-terminal fragment
[Hoxhaj G, Dissanayake K, MacKintosh C (2013) Effect of IRS4 Levels on PI 3-Kinase Signalling. PLoS ONE 8(9): e73327. https://doi.org/10.1371/journal.pone.0073327]

## Slide 3
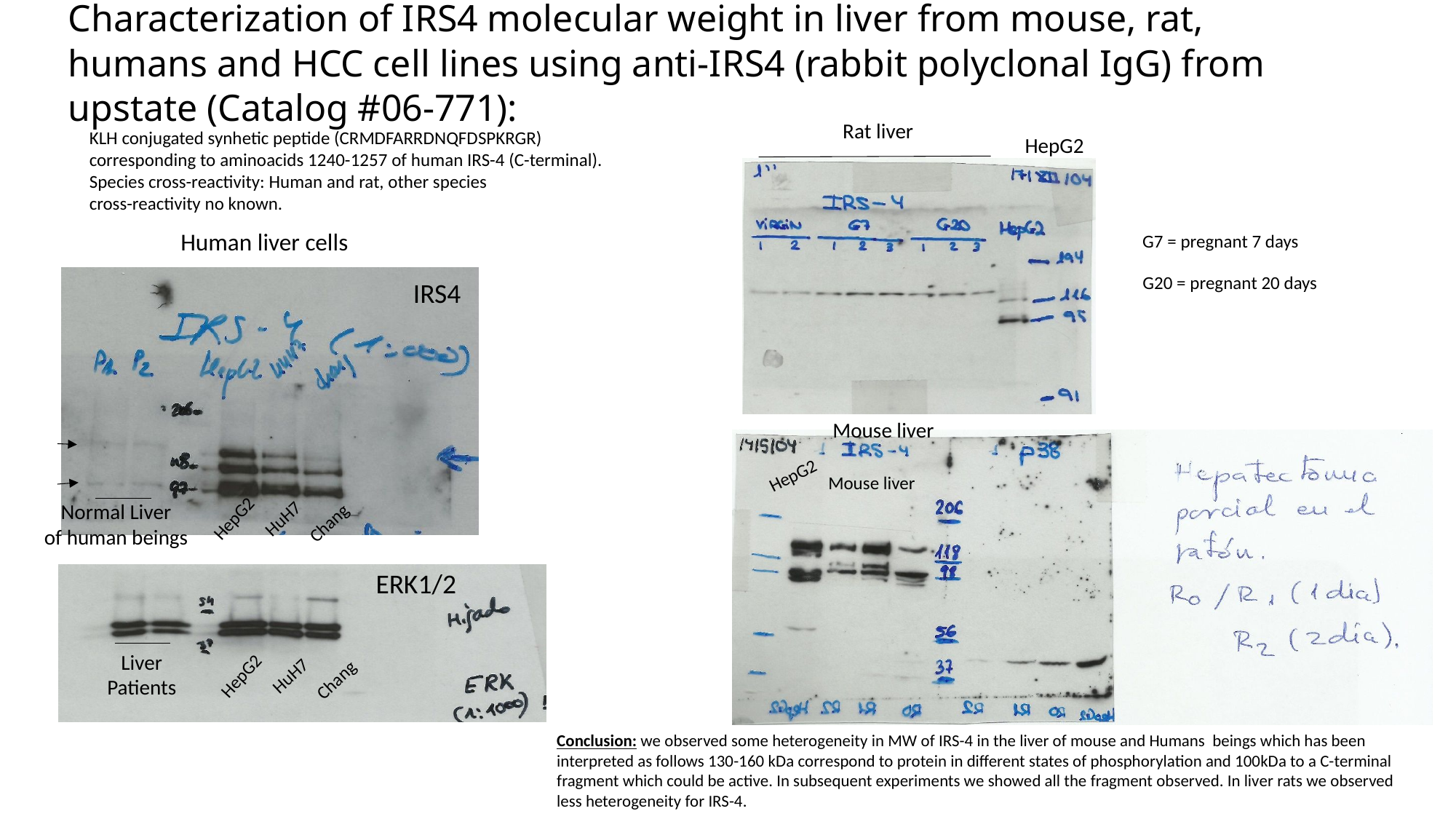

# Characterization of IRS4 molecular weight in liver from mouse, rat, humans and HCC cell lines using anti-IRS4 (rabbit polyclonal IgG) from upstate (Catalog #06-771):
Rat liver
HepG2
KLH conjugated synhetic peptide (CRMDFARRDNQFDSPKRGR)
corresponding to aminoacids 1240-1257 of human IRS-4 (C-terminal).
Species cross-reactivity: Human and rat, other species
cross-reactivity no known.
Human liver cells
G7 = pregnant 7 days
G20 = pregnant 20 days
IRS4
Normal Liver
of human beings
HuH7
HepG2
Chang
ERK1/2
Liver
Patients
HuH7
HepG2
Chang
Mouse liver
HepG2
Mouse liver
Conclusion: we observed some heterogeneity in MW of IRS-4 in the liver of mouse and Humans beings which has been interpreted as follows 130-160 kDa correspond to protein in different states of phosphorylation and 100kDa to a C-terminal fragment which could be active. In subsequent experiments we showed all the fragment observed. In liver rats we observed less heterogeneity for IRS-4.

## Slide 4
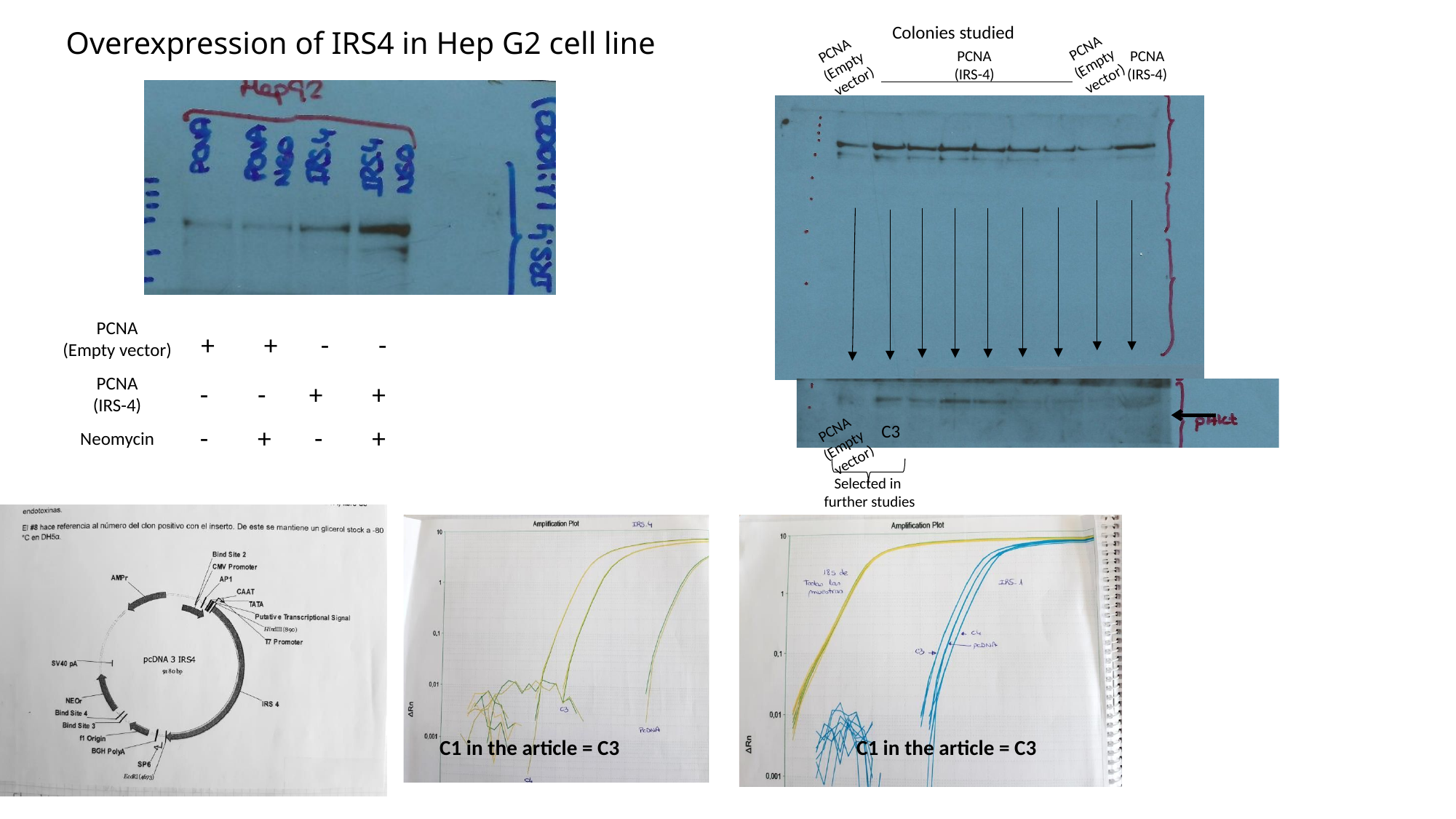

# Overexpression of IRS4 in Hep G2 cell line
Colonies studied
PCNA
(Empty
 vector)
PCNA
(Empty
 vector)
PCNA
(IRS-4)
PCNA
(IRS-4)
PCNA
(Empty
 vector)
C3
Selected in
further studies
PCNA
(Empty vector)
+ + - -
PCNA
(IRS-4)
- - + +
- + - +
Neomycin
C1 in the article = C3
C1 in the article = C3

## Slide 5
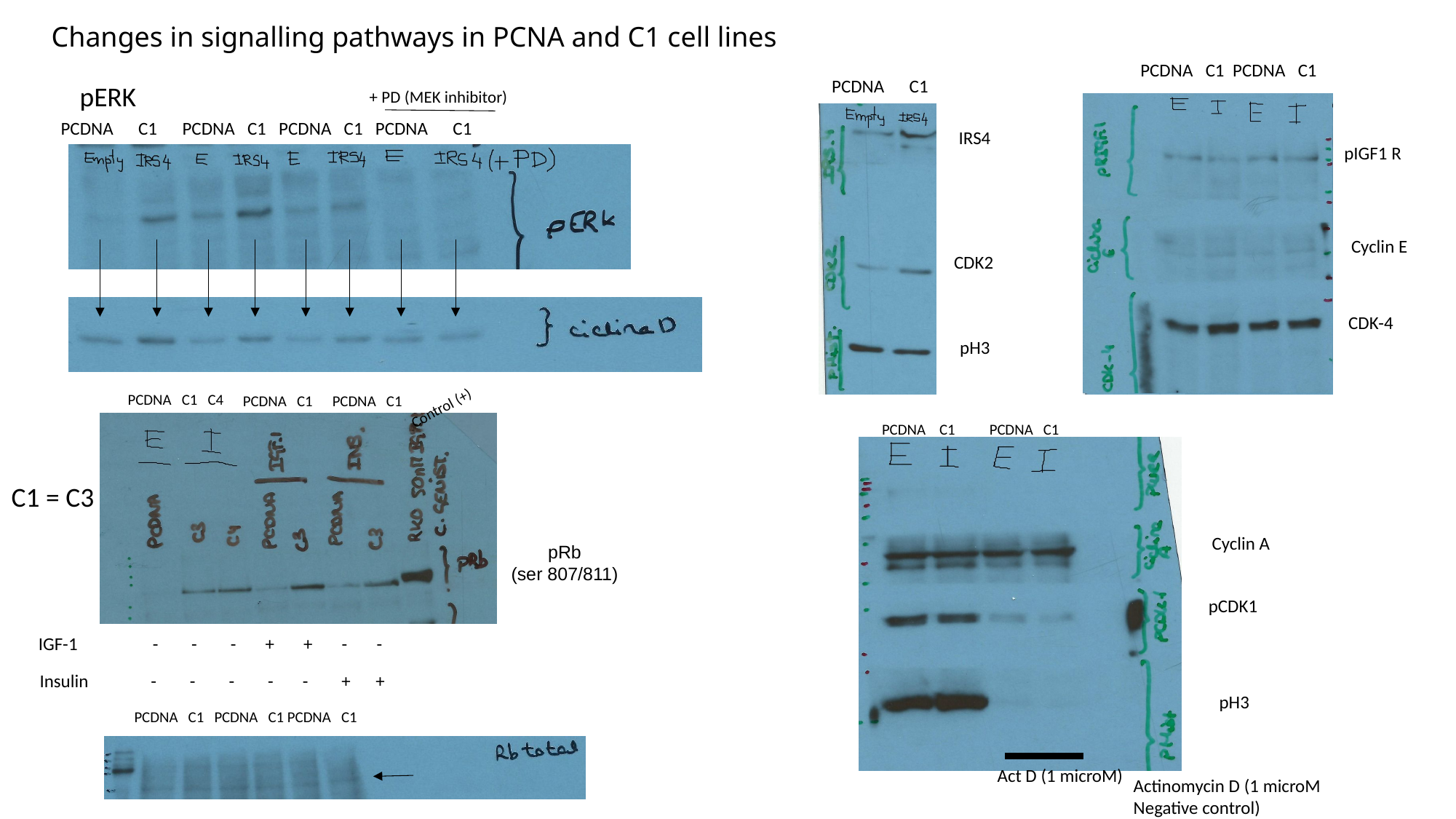

# Changes in signalling pathways in PCNA and C1 cell lines
PCDNA C1 PCDNA C1
pIGF1 R
Cyclin E
CDK-4
PCDNA C1
IRS4
CDK2
pH3
pERK
+ PD (MEK inhibitor)
PCDNA C1 PCDNA C1 PCDNA C1 PCDNA C1
PCDNA C1 C4
PCDNA C1
PCDNA C1
Control (+)
PCDNA C1 PCDNA C1
Act D (1 microM)
C1 = C3
Cyclin A
pRb
(ser 807/811)
pCDK1
IGF-1 - - - + + - -
Insulin - - - - - + +
pH3
PCDNA C1 PCDNA C1 PCDNA C1
Actinomycin D (1 microM
Negative control)

## Slide 6
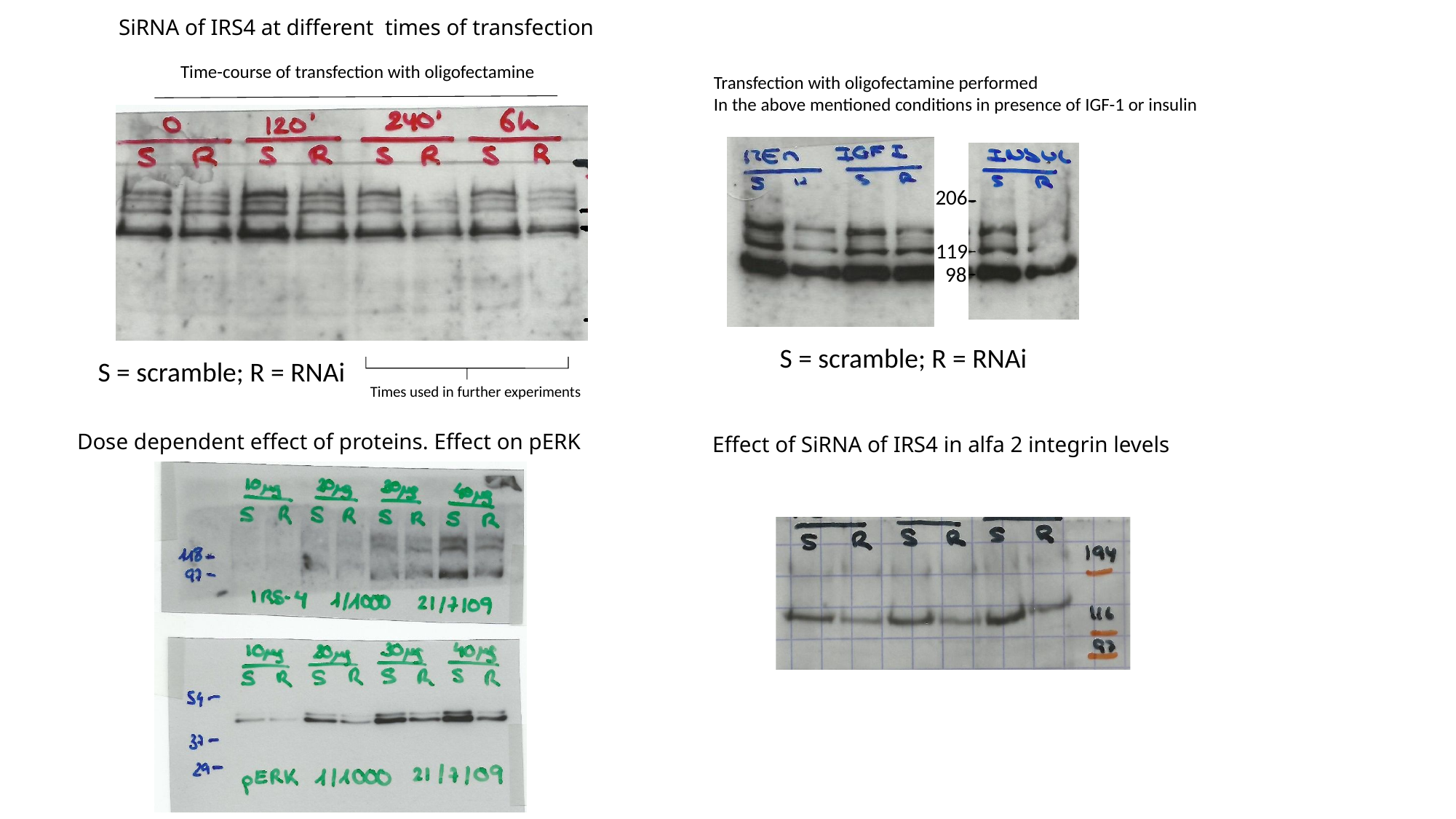

# SiRNA of IRS4 at different times of transfection
Time-course of transfection with oligofectamine
Transfection with oligofectamine performed
In the above mentioned conditions in presence of IGF-1 or insulin
206
119
98
S = scramble; R = RNAi
S = scramble; R = RNAi
Times used in further experiments
Dose dependent effect of proteins. Effect on pERK
Effect of SiRNA of IRS4 in alfa 2 integrin levels

## Slide 7
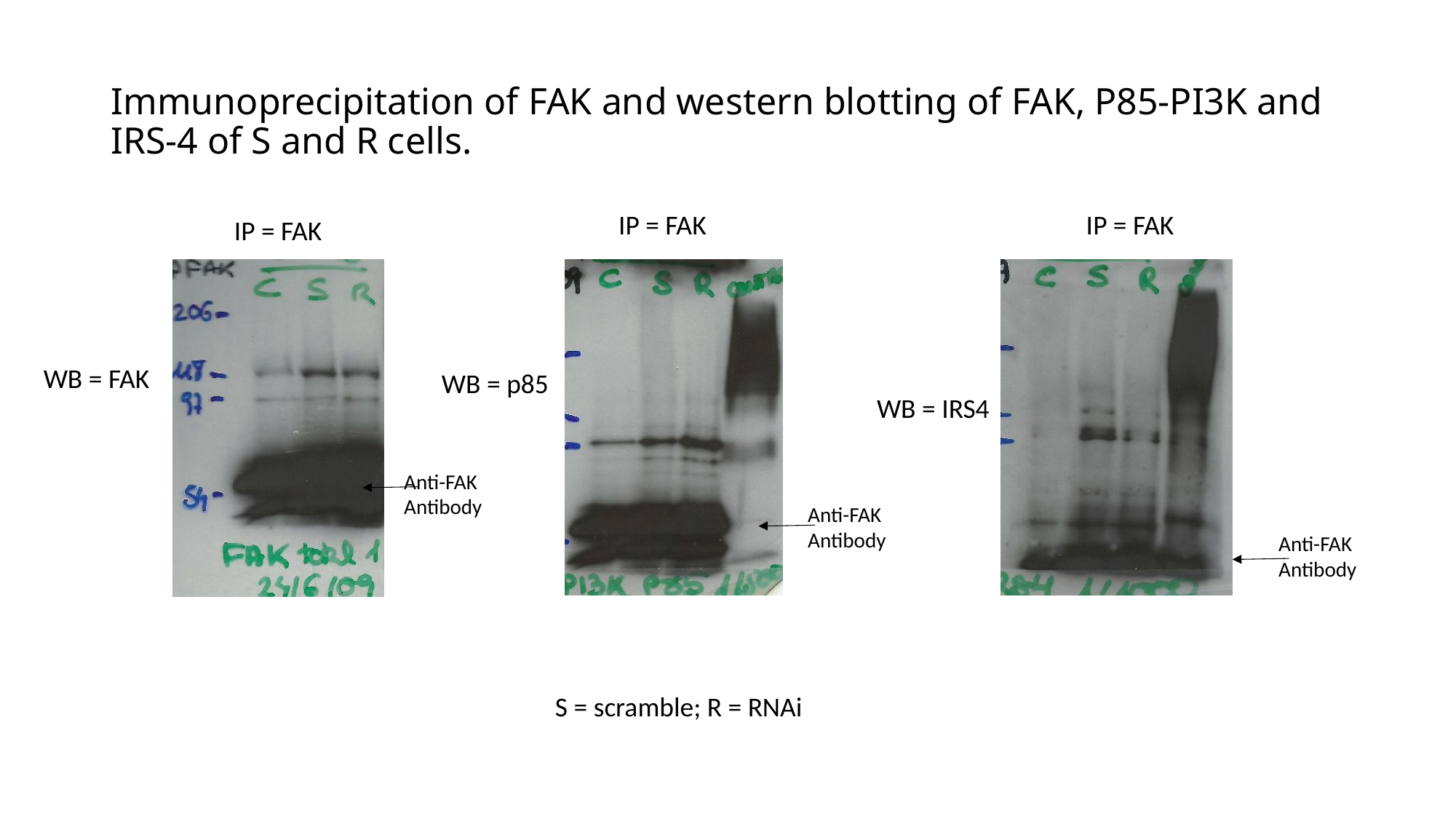

# Immunoprecipitation of FAK and western blotting of FAK, P85-PI3K and IRS-4 of S and R cells.
IP = FAK
IP = FAK
IP = FAK
WB = FAK
WB = p85
WB = IRS4
Anti-FAK
Antibody
Anti-FAK
Antibody
Anti-FAK
Antibody
S = scramble; R = RNAi

## Slide 8
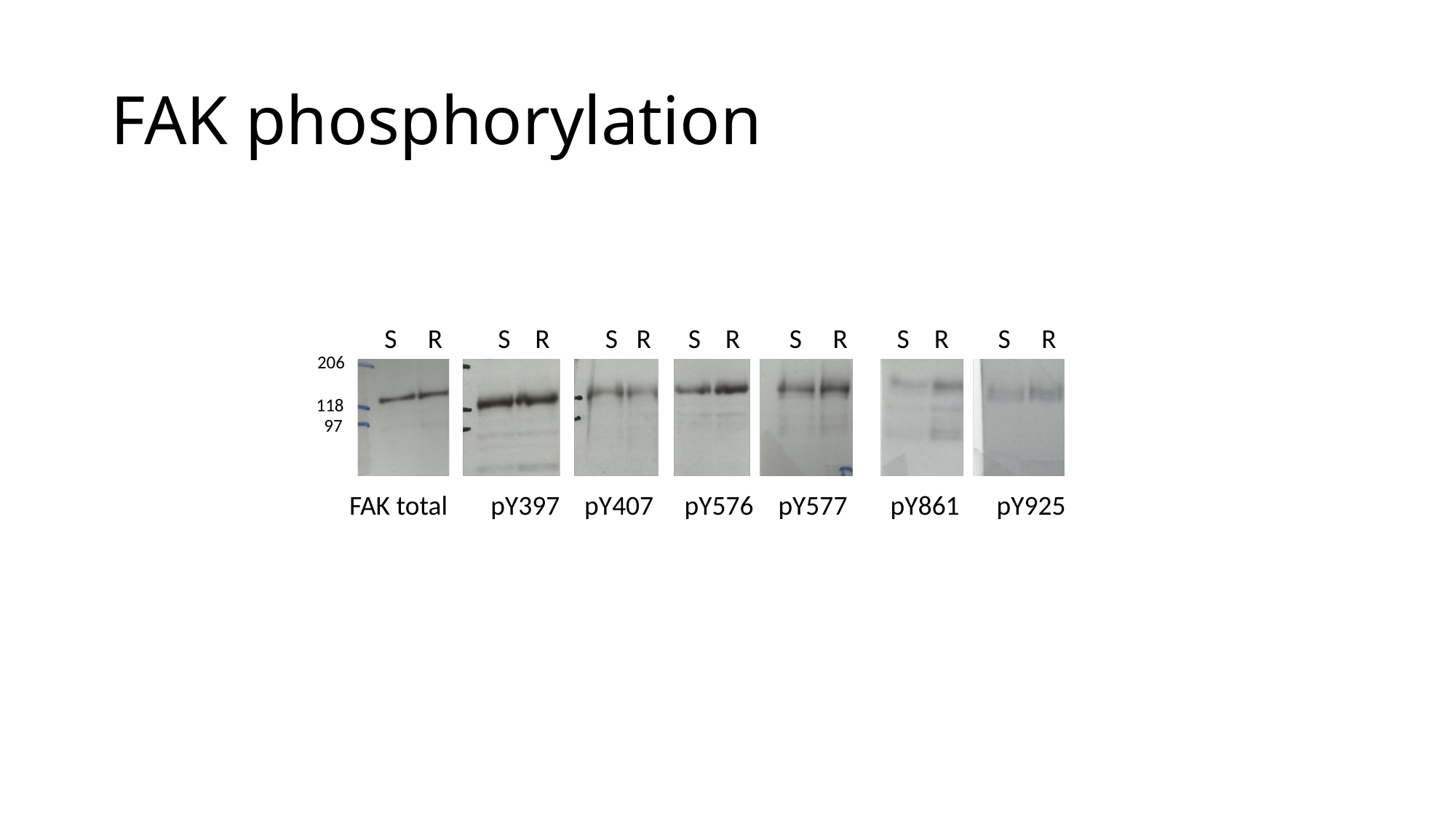

# FAK phosphorylation
S R S R S R S R S R S R S R
FAK total pY397 pY407 pY576 pY577 pY861 pY925
206
118
97
